# PET-Based Volumetric Biomarkers for Risk Stratification of Non-Small Cell Lung Cancer Patients

**DOI:** 10.3390/diagnostics11020210

**Published:** 2021-01-30

**Authors:** Sara Pellegrino, Rosa Fonti, Alessandro Pulcrano, Silvana Del Vecchio

**Affiliations:** 1 Department of Advanced Biomedical Sciences, University “Federico II”, 80131 Naples, Italy; sara.pellegrino@unina.it (S.P.); alessandro.pulcrano@unina.it (A.P.); 2 Institute of Biostructures and Bioimages, National Research Council, 80145 Naples, Italy; rosa.fonti@ibb.cnr.it

**Keywords:** 18F-FDG PET/CT, non-small cell lung cancer, metabolic tumor volume, total lesion glycolysis, prognosis, immunotherapy

## Abstract

Despite the recent advances in lung cancer biology, molecular pathology, and treatment, this malignancy remains the leading cause of cancer-related death worldwide and non-small cell lung cancer (NSCLC) is the most common form found at diagnosis. Accurate staging of the disease is a fundamental prognostic factor that correctly predicts progression-free (PFS) and overall survival (OS) of NSCLC patients. However, outcome of patients within each TNM staging group can change widely highlighting the need to identify additional prognostic biomarkers to better stratify patients on the basis of risk. 18F-FDG PET/CT plays an essential role in staging, evaluation of treatment response, and tumoral target delineation in NSCLC patients. Moreover, a number of studies showed the prognostic role of imaging parameters derived from PET images, such as metabolic tumor volume (MTV) and total lesion glycolysis (TLG). These parameters represent three-dimensional PET-based measurements providing information on both tumor volume and metabolic activity and previous studies reported their ability to predict OS and PFS of NSCLC patients. This review will primarily focus on the studies that showed the prognostic and predictive role of MTV and TLG in NSCLC patients, addressing also their potential utility in the new era of immunotherapy of NSCLC.

## 1. Introduction

Lung cancer is the leading cause of cancer-related death for both men and women with a higher incidence in developed countries [1]. Depending on the histotype, two main forms of lung cancer were identified: non-small cell lung cancer (NSCLC) that accounts for approximately 85% of lung cancer cases at diagnosis, and small cell lung cancer with a more aggressive biological behavior.

Despite the recent advances in screening, diagnosis, molecular pathology, and therapeutic strategies, the outcome of NSCLC patients remains poor [2]. Many factors have been used to predict the biological behavior of NSCLC including stage, weight loss, performance status, proliferation, histology, and molecular markers. To date, the most important prognostic factor remains stage of the disease at diagnosis that correctly predicts progression-free (PFS) and overall survival (OS) of NSCLC patients thus guiding decisions on subsequent therapy. However, although stage is effectively used for planning the therapeutic regimen in NSCLC patients, a wide variation of treatment responses and overall outcome were observed among patients with the same stage. This means that within each class of TNM staging, an additional prognostic stratification could identify homogeneous groups of patients with the same risk of progression and death. To this end, in addition to stage, other prognostic biomarkers should be identified to better stratify patients on the basis of risk thus allowing risk-adapted treatment strategies.

18F-labeled 2-deoxy-D-glucose Positron Emission Tomography/Computed Tomography (18F-FDG PET/CT) is a recognized essential tool for staging [3,4], evaluation of treatment response [5] and tumoral target delineation in NSCLC patients [6,7]. In addition, several studies showed that PET imaging findings are predictive of PFS and OS in these patients [8,9,10]. In fact, several imaging parameters were derived from PET images and correlated with other prognostic variables and clinical outcome.

The most common and simplest PET derived parameter is the Standardized Uptake Value (SUV), a semiquantitative measurement of FDG uptake, representing the activity within a region of interest (ROI) normalized for body weight and injected dose. Maximum SUV (SUVmax) indicates the highest FDG uptake in a single voxel within an ROI and is currently used in clinical practice as an index of FDG uptake being easy to calculate and operator-independent. A number of studies showed the predictive and prognostic value of SUVmax determined in primary tumors of NSCLC patients at initial diagnosis [11,12,13,14,15,16,17,18], after induction therapy [19,20,21], and in post-treatment evaluation [22,23]. A major limitation of SUVmax is that it provides a semiquantitative estimate of FDG activity in a single voxel of a tumor mass and therefore it may not be representative of the metabolic status of the whole tumor. To overcome this limitation, PET-based volumetric imaging parameters such as metabolic tumor volume (MTV) and total lesion glycolysis (TLG) have been proposed and their association with clinical outcome of NSCLC patients has been tested. MTV and TLG represent three-dimensional parameters including information on both tumor volume and metabolic activity. MTV represents the volume inside an operator- or algorithm-defined ROI that segments the metabolically active component of the tumor. TLG is calculated as the product of MTV and the correspondent value of mean SUV (SUVmean). A number of studies evaluated the prognostic role of MTV and TLG measured in primary tumors and showed that these volume-based parameters can better predict PFS and OS as compared to SUVmax [24,25,26]. Furthermore, MTV and TLG can be determined not only in primary tumors but also in regional lymph nodes and metastatic sites thus providing the total metabolic tumor burden of each patient. The sum of MTV and TLG of all lesions in a patient will reflect the volumetric extension of metabolically active disease and the aggressiveness of the tumor thus allowing a better stratification of patients within each stage and adoption of risk-adapted therapy.

Beyond their prognostic value, MTV and TLG may be predictive of treatment response [27,28]. Changes in MTV and TLG during chemotherapy were reported to be associated with overall tumor response at the end of treatment [29]. Finally, MTV and TLG were found clinically helpful in tumor delineation for radiotherapy planning [30].

The present article will provide an overview of the studies showing the prognostic and predictive role of MTV and TLG in NSCLC patients preceded by a recapitulation of the current methods for determination of these volume-based parameters. Finally, the role of MTV and TLG in the new era of immunotherapy of lung cancer will be also evaluated.

## 2. Methodological Aspects of Tumor Volume Delineation

Several methods have been proposed for delineating tumor borders on PET images, using manual, semiautomatic and automatic approaches [31,32,33,34,35]. Tumor boundaries can be manually drawn by a nuclear medicine physician, a radiologist, or a radiation oncologist based on visual perception of the tumor border, and the volume of that region is calculated to obtain MTV. This manual method has some limitations in fact the determination of the tumor boundary depends on both the experience of the physician and the contouring protocol used [36].

Alternatively, tumor boundaries can be delineated by automatic or semiautomatic methods using a fixed pre-defined threshold, and all voxels with SUV above the threshold are assigned to tumor and all SUV below the threshold are considered part of the background. This approach has been extensively used for tumor volume delineation on PET images since it has been reported to reduce the inter- and intra-observer variability of the measurement.

The threshold can be an absolute SUV value (fixed absolute threshold) or can be expressed as a percentage of SUVmax within the tumor (fixed relative threshold). Previous studies using an absolute fixed threshold method reported different values of SUV as potential absolute thresholds. However, the most widely accepted threshold is an SUV value of 2.5 based on the assumption that background activity is around that value [37]. This method has several limitations, in particular, well-differentiated tumors with low FDG uptake can remain below the selected threshold, precluding the possibility to measure MTV in these tumors. On the other hand, a significant overestimation of MTV can occur in lesions that are near to areas with high physiological FDG uptake [38].

When using a relative fixed threshold in clinical settings, the most common threshold chosen for tumor delineation is 40–43% of SUVmax [39]. Even in this case, it is possible to have an under and an overestimation of MTV. For instance, despite a high SUVmax value, tumor volume may be underestimated due to the heterogeneous distribution of SUV values and hence the presence of many voxels with SUV values lower than the threshold. On the contrary, if the threshold is too low, the delineated volume may include part of the background causing an overestimation of tumor volume [30].

To find a more accurate threshold and to overcome these limitations, a background threshold method has been proposed. Using this method, an ROI is drawn in the liver or in the mediastinal blood pool and then background SUV is measured. Generally, the threshold is defined as SUV mean plus 1 or 2 standard deviation (SD) of the background [27,40]. In addition to the use of liver or blood pool as the background reference region, the background immediately surrounding the tumor can be used to delineate tumor volume [41]. Using this approach the mode of SUV distribution in the ROI is used to describe the background uptake. Based on the assumption that background activity has a Gaussian distribution, this Gaussian distribution can be subtracted from the original ROI to obtain tumor segmentation. Most heterogeneous tumors can be analyzed for determination of MTV and TLG using this method of background-subtracted volume (BSV).

To overcome the limitations of threshold methods, more advanced algorithms have been developed to delineate tumor boundaries using adaptive thresholds. Among them, gradient-based methods define tumor borders by exploiting the image gradient that exists between the high SUV in tumor cells and the lower SUV in adjacent non-tumor tissues [42]. In fact, these methods identify tumor edges based on a sharp change in count levels at the tumor border. Other algorithm-based methods include classifier-based methods and statistical methods. In particular, fuzzy c-means (FCM) algorithm and fuzzy locally adaptive Bayesian (FLAB) algorithm have been used in lung cancer [43,44].

To date, although several segmentation methods have been proposed, a standardization has not been achieved yet and more validation studies are needed. Nevertheless, MTV was revealed to be a strong predictor of prognosis irrespective of the method used for measurement in many types of malignancies [45,46].

## 3. The Prognostic Role of Volume-Based PET Parameters in Primary NSCLC

Many studies have evaluated the prognostic role of volume-based PET parameters such as MTV and TLG measured in primary tumors of NSCLC patients (Table 1).

Im et al., conducted a comprehensive systematic review of the studies evaluating these parameters and their prognostic role in patients with lung cancer [55]. They reported that MTV and TLG of primary tumors were strong prognostic factors of outcome in patients with both early and advanced NSCLC. In fact, patients with high MTV or TLG showed a worse prognosis than patients with low MTV or TLG values. Moreover, the prognostic value of MTV and TLG remained significant regardless of TNM stage, methods for tumor delineation, and selection of cut-off values in the survival analysis.

In a meta-analysis, including 5807 patients, Liu et al. [56] found that higher values of SUVmax, MTV, and TLG predicted a higher risk of disease recurrence or death in NSCLC patients who were candidates for surgery. They suggested the use of 18F-FDG PET/CT to select patients with higher risk of disease recurrence or death that may benefit from additional treatment. The positive association remained statistically significant across analyses in which patients are stratified by stage, pathology, and cut-off values.

Davison et al. [47] determined MTV and TLG values on baseline PET using a gradient-based method in 39 patients with NSCLC at various stages of disease. When survival was analyzed at the end of follow-up, MTV was significantly greater in patients who died than in patients who survived whereas no significant difference was found in TLG and SUVmax of those alive or dead. When setting the survival time earlier, at 12 months, both MTV and TLG were significantly greater in those who died than in those who survived. Survival analysis showed that OS was significantly better in patients showing MTV and TLG lower than their median value (9.7 mL and 74 g, respectively) and, at multivariate analysis, only MTV was an independent predictor of 12 months survival.

Hyun et al. [40] evaluated the prognostic role and predictive performance of volume-based parameters in 529 patients with early-stage NSCLC who underwent preoperative 18F-FDG PET/CT. SUVmax, MTV, and TLG of the primary tumors were obtained. They demonstrated that volume-based parameters were important independent prognostic factors for survival in addition to the pathological TNM stage and predicted survival more accurately than SUVmax alone. In particular, the estimated 5-year OS rates were 89.0% for patients with MTV ≤16 cm^3^ and 66.7% for those with MTV >16 cm^3^. Similarly, the estimated 5-year disease-free survival (DFS) rates were 67.4% and 52.4% for patients with MTV lower or higher than the cut-off value, respectively. In addition, the authors reported that the estimated 5-year OS rates were 90.3% for patients with TLG ≤70 g and 66.0% for those with TLG >70 g. Moreover, the estimated 5-year DFS rates were 69.9% and 50.2% for patients with TLG lower or higher than the cut-off value, respectively. Furthermore, all PET parameters in adenocarcinomas tended to be significantly lower than in nonadenocarcinomas and histology was a significant variable in the prediction of OS at univariate analysis.

Anwar et al. [48] studied 49 patients with pathologically proven stage I NSCLC who underwent 18F-FDG PET/CT at baseline followed by complete surgical resection of the tumor. Their main purpose was to obtain PET parameters capable of identifying patients at high risk of recurrence thus requiring further post-operative treatment. Their results showed that baseline SUVmax, MTV, and TLG were statistically significant prognostic factors in completely resected stage I NSCLC. Notably, MTV was more accurate than SUVmax in predicting recurrence with 89% sensitivity and 73% specificity. The authors demonstrated that one-year DFS rate in patients with MTV below the cut-off of 6.6 mL obtained by ROC curve analysis was 96%, while in those with MTV above the cut-off was 80%. Moreover 3-year DFS was 96% and 36% in patients with MTV lower or higher than the cut-off, respectively.

These findings were in agreement with the results of another study conducted by Dosani et al. [49] including 134 patients with inoperable early-stage NSCLC treated using stereotactic ablative radiotherapy (SABR) with curative intent. MTV appeared to be prognostic of local control (LC) and OS in this patient cohort. In fact, when patients were dichotomized into low-MTV and high-MTV subgroups based on the median MTV of 2.4 mL, those in the high-MTV group had worse outcome (26.9 vs. 48.3 months). Moreover, at 2 years, LC was 100% in the low-MTV group and 82.7% in the high-MTV group. Finally, no relationship of histologic type with LC was evident in this study.

An additional study by Yanarates et al. [50] evaluated the prognostic value of metabolic parameters determined in primary tumors of 258 patients with advanced-stage lung adenocarcinoma who had undergone pretreatment 18F-FDG PET/CT. At follow-up, they found that OS was significantly better in patients with MTV lower than the optimal cut-off value of 5.7 mL calculated by ROC curve analysis (27 vs. 14 months). Similarly, OS was significantly better in patients with TLG lower than the cut-off of 49.4 g (24 vs. 13 months). However, no significant relationship was found between volumetric PET parameters and PFS. Moreover, in this study, OS and PFS were not significantly different in patients with or without EGFR mutations.

Kim et al. [51] investigated the predictability of occult lymph node metastasis (OLM) using metabolic parameters on pretreatment 18F-FDG PET/CT in squamous cell non-small cell lung carcinoma (SC-NSCLC) patients who were clinically node-negative before surgery. At multivariate analysis, high values of SUVmax and MTV showed an association with an increased risk of OLM. SUVmax, MTV, and TLG cutoff levels were determined by ROC curve analysis, and the best discriminative values for predicting OLM were 8.8, 18.9 cm^3^ and 88.4 g, respectively. Thereby, the authors demonstrated that there was a significantly higher rate of OLM in patients with SUVmax >8.8, MTV >18.9 cm^3^, or TLG of >88.4 g, as compared to those with SUVmax ≤8.8, MTV ≤18.9 cm^3^, or TLG ≤88.4 g, respectively. However, the analysis of the area under the ROC curve showed that MTV (AUC 0.758) had a better predictive performance than SUVmax (AUC 0.712) and TLG (AUC 0.737) for the prediction of OLM.

Another study by Park et al. [52] included 139 patients with small-size peripheral NSCLC without lymph node metastasis. Since these patients can be optimal candidates for sublobar resection, the main purpose of the study was to identify predictors of occult lymph node metastasis (OLM) using 18F-FDG PET/CT. MTV showed a better predictive performance than other PET parameters and was proposed as a possible indicator for sublobar resection in clinically node-negative small-sized NSCLC. In fact, ROC curve analysis showed that AUC of MTV for occult lymph node metastasis (N1 and N2) was higher as compared to AUCs of SUVmax and TLG and the optimal cut-off values were 3.250, 3.055 mL, and 9.829 g for SUVmax, MTV, and TLG, respectively. Moreover, histology, grade, and T stage were not significantly associated with the presence of OLM.

Additional studies evaluated the ability of volume-based PET parameters changes during treatment to predict outcome in NSCLC patients. For instance, Roengvoraphoj et al. [53] studied 65 patients with inoperable locally advanced NSCLC (stage IIIA/B, TNM 7th edition) treated with definitive chemoradiotherapy in order to identify PET-based parameters with a prognostic value during multimodality treatment. Their study evaluated the role of MTV changes before, during, and after chemoradiotherapy (CRT) in primary tumors. These authors found that patients with pre-MTV >63 cm^3^ and those with post-MTV >25 cm^3^ both showed significantly worse outcomes. Moreover, their results indicated that an MTV reduction of at least 15% from the third week (mid-MTV) to the end of CRT (post-MTV) significantly correlated with an improved outcome.

Another study by the same authors [54] extensively investigated the prognostic value of MTV reduction after treatment in a homogeneous cohort of 60 patients with inoperable stage III NSCLC treated with definitive chemoradiotherapy. The authors reported that an MTV reduction of at least 80% after CRT indicated a complete or major metabolic response and was associated with a significantly improved patient outcome. In fact, at multivariate analysis, significant predictors of survival included ECOG performance status along with complete and major metabolic response assessed by ΔMTV ≥80%. A moderate metabolic response did not correlate with improved outcome.

## 4. The Prognostic Role of Volume-Based PET Parameters in All Metabolically Active Lesions of NSCLC

Volume-based PET parameters can be measured not only in primary tumors but also in involved lymph nodes and distant metastases. The total or whole-body MTV or TLG are obtained by summing the MTV or TLG values of all measurable lesions in a patient. Therefore, these volumetric parameters will reflect the entire metabolic tumor burden and may have a significant clinical impact on the management of NSCLC patients. A number of studies evaluated indeed the prognostic role and the stratification power of these parameters (Table 2).

Bazan et al. [57] reported that pretreatment whole-body MTV (MTV-pre) predicted OS in a group of uniformly treated patients with stage III NSCLC, independently of other prognostic factors. In addition, their results indicated that patients with high whole-body MTV, if treated with a more aggressive regimen including high-dose radiation therapy, may have improved survival. In particular, patients with whole-body MTV-pre >32 mL (the median value) had significantly worse OS than patients with whole-body MTV-pre ≤32 mL (14.8 vs. 29.7 months). Moreover, in the population with whole-body MTV-pre >32, patients who received ≤60 Gy had worse outcome than those who received >60 Gy. At univariate analysis, higher whole-body MTV-pre was associated with a worse LC. However, although whole-body MTV-pre was predictive of LC at 6 months, the same parameter was no longer prognostic of LC at 1 year. Furthermore, patients with measurable post-treatment whole-body MTV (MTV-post) had significantly worse OS than patients with no residual whole-body MTV-post.

Another study including only NSCLC patients in stage III reported the use of whole-body MTV to stratify patients for the adoption of the most appropriate therapeutic strategy [58]. The authors found that volume-based PET parameters may help to choose whether a patient in stage IIIA should receive a more aggressive treatment than that for stage IIIB or a less intensive regimen than that for stage IIB. Therefore, they divided the stage IIIA patients into two subgroups: group IIIA(−) with whole-body MTV ≤29.2 mL (44.5% of patients) and group IIIA(+) with whole-body MTV >29.2 mL (55.5%). Using this cut-off value, patients with stage IIIA(−) and stage IIIA(+) had a survival profile not significantly different from patients with stage IIB and stage IIIB, respectively. Kaplan–Meier curves also showed a worse OS for stage IIIA patients with whole-body MTV >29.2 mL as compared to those with whole-body MTV ≤29.2 mL (1.47 vs. 2.93 years).

An additional study by Ventura et al. [59] investigated the prognostic role of metabolic parameters in 193 patients undergoing curative surgery with primary lung adenocarcinoma (ADC) with the aim to identify new prognostic markers suitable for further stratification of these patients. In this study, univariate analysis showed that SUVmax, MTV, and TLG measured on preoperative 18F-FDG-PET/CT had a significant prognostic value in patients with lung ADC candidate to surgical resection. By ROC curve analysis, AUC and cut-off values of MTV were 0.647 and 8.15 mL, respectively, whereas AUC and cut-off values of TLG were 0.691 and 21.85 g, respectively. In patients with MTV less than 8.15 mL, the mean OS was 79.7 months whereas patients with MTV greater than 8.15 mL showed a mean OS of 56.7 months. The mean OS was 85.5 and 52.7 months in patients with a TLG lower or higher than 21.85 g, respectively. Moreover, TLG appeared to be an independent prognostic indicator at multivariate analysis. Moreover, this study showed that the combination of metabolic parameters with clinical and biological markers could further stratify patients with lung ADC, allowing therapeutic strategies tailored on an individual basis.

A study conducted by Liao et al. [24] evaluated the prognostic role of volumetric parameters in 169 nonsurgical NSCLC patients. MTV and TLG of whole-body tumor, of primary tumor, of nodal metastases, and of distant metastases measured on baseline 18F-FDG PET/CT were found to be prognostic factors independently of clinical stage. In particular, when Kaplan–Meier curves were constructed after creating three roughly equal-sized groups using tertiles, the median OS was 19.9, 10.0, and 6.6 months, respectively using whole-body MTV while it was 17.4, 9.0, and 8.1 months, respectively using whole-body TLG.

**Figure 1:**
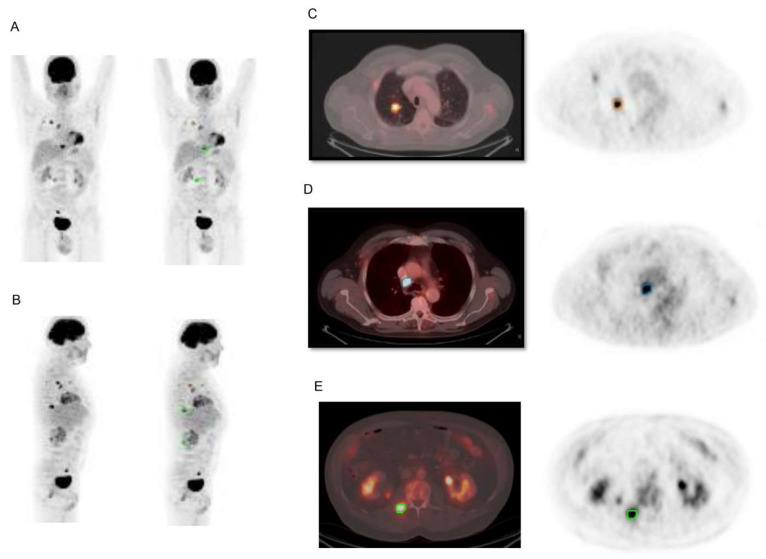
Representative images of tumor segmentation for determination of volume-based parameters on 18F-FDG PET/CT in a 59-years-old patient with stage IVB lung adenocarcinoma. Maximal intensity projection images are shown in panels (**A**) and (**B**). Transaxial PET images and fusion images of co-registered PET and CT are showed in panels (**C**–**E**). Tridimensional regions of interest were drawn around primary tumor, lymph nodes and bone metastases, and segmentation was performed using an automated contouring program setting a threshold of 2.5 for SUVmax. Examples of segmentation of primary lung tumor, lymph node, and bone metastasis are provided in panels (**C**–**E**). Whole-body MTV was 47.27 mL and whole-body TLG was 115.36 g. OS of the patient was 9 months.

Pellegrino et al. [60] studied 65 patients with NSCLC in all stages of disease showing that whole-body MTV and TLG derived from 18F-FDG PET/CT scan were useful prognostic factors to predict survival in patients with NSCLC. Whole-body MTV was indeed an independent prognostic parameter for OS providing additional information regardless of the stage and whole-body TLG was a predictor of PFS in NSCLC patients independently from the stage. In particular, ROC curve analysis identified a cut-off level of 54.7 g (AUC = 0.76) for whole-body TLG that was able to discriminate patients with and without progression. Using Kaplan–Meier analysis and long-rank testing, patients with whole-body TLG ≤54.7 g showed a significantly prolonged PFS as compared to patients with TLG >54.7 g (28 vs. 11 months). Similarly, a threshold was determined for total MTV and the best discriminative value between patients who had died and survivors was 9.5 mL. OS was significantly better in patients with total MTV ≤9.5 mL as compared to those with total MTV >9.5 mL (32 vs. 15 months). Both MTV and TLG could be determined on each component of the TNM system (Figure 1) allowing further stratification of patients within the same stage and subsequent adaptation of therapy in individual patients.

Another study conducted by Chen et al. [61] evaluated the prognostic role of whole-body TLG showing that it could be a promising tool for stratifying patients with NSCLC for risk-adapted therapies. Using ROC analysis, a cut-off of 655 g was determined for whole-body TLG to yield a specificity of 95%. Patients with whole-body TLG >655 g had poorer PFS and OS than those with whole-body TLG ≤655 g.

Vanhoveet et al. [62] studied 105 NSCLC patients in all stages of disease evaluating whole-body MTV and whole-body TLG. In this study, OS of patients with TLG ≥93.4 g (the median value) was 11 months whereas patients with TLG <93.4 g had an OS of 37 months. Moreover, PFS was significantly prolonged in patients with whole-body TLG <93.4 g (27months) as compared to those with whole-body TLG ≥93.4 g (8 months). In the multivariate model, gender, stage, whole-body MTV and whole-body TLG were independent prognostic factors for OS, while only TNM stage and whole-body TLG were prognostic factors for PFS. In contrast, TLG of the primary tumor had no significant role in the prediction of outcome.

The relative stratifying power of whole-body MTV and cTNM staging used alone or in combination, was evaluated in 278 NSCLC patients [63]. Whole-body MTV was found to be an independent and statistically significant predictor of OS in NSCLC patients. In fact, patients with an MTV lower than the cut-off of 49.5 mL had an OS (56.31 months) significantly different from those with a higher MTV (21.66 months). Moreover, its combination with cTNM stage had a higher prognostic value as compared to that of cTNM stage alone.

Pu et al. [64] developed and validated a novel whole-body MTV risk stratification system that could be used for further NSCLC pretreatment assessment and for refining patient’s treatment decisions. The proposed system used whole-body MTV quartiles to define MTV risk classes similar to the definition of stages in the TNM system. Three cut-off points of whole-body MTV at 10.0, 53.4, and 155.0 mL were derived based on the quartiles that identified four MTV strata. Their results showed that the whole-body MTV risk classification and TNM stages described different aspects of tumor activity as there was a large variation of whole-body MTV within individual TNM stages and substages. Therefore, whole-body MTV risk stratification system provided additional information that could guide the selection of treatment.

An additional study conducted by Chin et al. [65] demonstrated the prognostic value of pretreatment metabolic imaging parameters in oligometastatic patients who underwent locally ablative treatment of all sites of disease. Their findings suggested that the metabolic burden of disease on pre-treatment 18F-FDG PET/CT scan could be a useful prognostic marker of survival after locally ablative high-dose radiation therapy in patients with oligometastatic NSCLC. In particular, patients with TLG values within the highest quartile (>86.8 units) had significantly shorter OS, as measured after their first RT course, compared to those with TLG values within the lower three quartiles. The median OS for this high-TLG group was 12.4 months while was 30.1 months for all other patients. Similarly, patients showing MTV values within the highest quartile (>17.8 mL) had shorter OS compared to those with MTV values within the lower three quartiles (13.0 vs. 27.8 months).

Kong et al. [29] examined the significance of mid-treatment tumor volume for survival prediction in patients with stage I-III NSCLC undergoing daily fractionated radiation. 18F-FDG PET/CT scans were obtained before radiotherapy and at mid-treatment after 40–50 Gy. Changes in mid-treatment PET-based volumetric parameters were significantly associated with survival. In patients who received conventional radiation doses (60–70 Gy) and had MTV reduction greater or less than the median value, the median survival times were 14 versus 22 months, respectively. In contrast, in patients who received mid-treatment PET-adapted radiation therapy and had MTV reduction greater or less than the median value, the corresponding median survival times were 33 versus 19 months, respectively.

Chen et al. [66] evaluated the prognostic value of volumetric metabolic parameters determined during and after radiation-based therapy in stage III NSCLC patients. They found that ΔTLG and ΔMTV, especially ΔTLG, determined during-RT had prognostic value. In fact, OS and PFS were significantly different in patients with ΔTLG higher and lower than the threshold (31 vs. 15 months OS and 17 vs. 8 months PFS) whereas ΔTLG, ΔMTV determined post-RT were not significative.

Therefore, 18F-FDG PET/CT scan performed during RT could be more useful than post-RT 18F-FDG PET/CT scan for risk stratification.

Xiao et al. [67], in their prospective study, quantified the metabolic tumor volumes (MTVs) in 18F-FDG PET/CT scan performed at baseline and in the late course of radiotherapy with the main purpose of reducing the risk of radiation toxicity and improving the quality of life of patients with NSCLC. Seventeen patients with stage II-III NSCLC who were treated with definitive conventionally fractionated RT were enrolled. Their study showed that PET-MTVs were significantly reduced at the time of approximately 40 Gy during RT (approximately two-thirds of the total dose) and late-course adaptive radiotherapy could be an effective method to reduce the dose volume to the organs at risk in patients with NSCLC.

## 5. Volume-Based PET Parameters in the Era of Immunotherapy

Many studies have investigated the potential role of volume-based PET parameters in NSCLC patients receiving targeted therapies and especially in those undergoing immunotherapy. Recently, immune checkpoint inhibitors have been introduced as an additional treatment option for NSCLC patients. Some of these agents specifically target the programmed cell death 1 receptor (PD-1) (pembrolizumab and nivolumab) or the programmed cell death-ligand 1 receptor (PD-L1) (atezolizumab), and have been approved by EMA and FDA for NSCLC treatment [68,69,70,71,72].

Kaira et al. [73] evaluated in their prospective study the role of volumetric parameters derived from PET/CT in predicting tumor response to nivolumab in NSCLC patients. Twenty-four patients were enrolled in their study. 18F-FDGPET/CT was carried out before and 1 month after nivolumab administration and SUVmax, MTV, and TLG were calculated on PET images. They showed that metabolic response assessed as changes of volumetric parameters (especially TLG) was closely associated with therapeutic response and survival after nivolumab administration.

The role of 18F-FDG PET/CT to predict response to immunotherapy was also evaluated in patients with metastatic lung cancer [74]. Thirty-two patients were enrolled and treated with nivolumab. Whole-body maximum standardized uptake value (SUVmaxwb), whole-body MTV, and whole-body TLG were obtained as the sum of SUVmax, MTV, and TLG in all metabolic lesions. They showed that the entire tumor burden evaluated by 18F-FDG PET/CT can be predictive of response to immunotherapy in patients with metastatic lung cancer.

Seban et al. [75] tested whether imaging biomarkers derived from FDG PET scan were associated with clinical outcomes in patients with advanced NSCLC treated with immune checkpoint inhibitors (ICIs). They included 109 patients with advanced NSCLC who underwent baseline FDG PET/CT before ICI; clinical, biological (including dNLR = neutrophils/[leukocytes minus neutrophils]), pathological, and PET parameters (tumor SUVmax, whole-body MTV) were evaluated. Baseline tumor burden (TMTV) on FDG PET/CT scans and inflammatory status (dNLR) were associated with poor OS for ICI treatment in advanced NSCLC patients and could be used together to improve the selection of appropriate candidates.

In another study, the authors [76] investigated the correlation between PET-based parameters and PD-L1 expression in tumor tissue, necrosis, and clinical outcome in patients receiving checkpoint inhibitor treatment. They studied 49 patients and evaluated SUVmax, SUVmean, MTV, and TLG obtained from 18F-FDG PET/CT images. The ratio of metabolic to morphological lesion volumes (MMVR) and its association with PD-L1 expression in each lesion were calculated. MMVR was inversely correlated with PD-L1 expression in tumor cells. Furthermore, PD-L1 expression and low MMVR were significantly correlated with clinical benefit. This study introduced MMVR as a new imaging biomarker highlighting its ability to noninvasively reveal increased PD-L1 expression in tumor thus predicting clinical benefit from checkpoint blockade in NSCLC.

Castello et al. [77] examined circulating tumor cells (CTC) and their association with metabolic parameters and clinical outcomes in NSCLC patients treated with immune checkpoint inhibitors. In their prospective study, 35 patients were enrolled and underwent a 18F-FDG PET/CT scan and CTC detection in peripheral blood samples obtained at baseline and 8 weeks after ICI initiation. Association of CTC count with clinical and metabolic characteristics was then studied. CTC count variation (∆CTC) was significantly associated with tumor metabolic response as assessed by the European Organization for Research and Treatment of Cancer (EORTC) criteria. At the first restaging, patients with a high tumor burden (high MTV and TLG) had a higher CTC count. The combination of mean CTC and median MTV at 8 weeks was associated with PFS and OS. Multivariate analysis identified CTC count at 8 weeks as an independent predictor for PFS and OS, whereas ∆MTV and maximum standardized uptake value variation (∆SUVmax) were predictive for PFS and OS, respectively. This study confirmed that CTC correlated with metabolic response during ICI. Moreover, elevated CTC count, along with metabolic parameters, were found to be prognostic factors for PFS and OS.

Wang et al. [78] investigated the correlation between metabolic status on 18F-FDG PET/CT and intra-tumor immunomarkers’ expression in NSCLC patients. Seven hundred and sixty-three patients were enrolled in the study to investigate the role of SUVmax in lung cancer and 122 tumor specimens were analyzed by immunohistochemistry (IHC) to evaluate intra-tumor immune cells and PD-L1 expression in tumor cells. The correlation between metabolic variables and the expression of tissue immune markers were analyzed. The authors showed that SUVmax was an independent prognostic factor in lung cancer patients. Furthermore, SUVmax values had significant variations in tumors with different epidermal growth factor receptor (EGFR) status (wild type vs. mutant type), high/low neutrophil-to-lymphocyte ratio (NLR), and high/low platelets-to-lymphocyte ratio (PLR). Moreover, MTV and TLG had a statistically significant correlation with progression-free survival and overall survival. Their study revealed an association between metabolic variables and immune cell expression in the tumor microenvironment and suggested that SUVmax determined on 18F-FDG PET/CT images could be used for selection of candidates for immunotherapy.

## 6. Conclusions

MTV and TLG are volume-based PET parameters providing a comprehensive evaluation of the viable tumor burden and biological aggressiveness of tumor. These parameters can be measured not only in primary tumors but also in all metabolically active metastatic lesions throughout the whole body. Although standardized methods for their measurements are still required, these volume-based parameters were revealed to be important prognostic factors independently of the method used. They may indeed identify different classes of risk in the same TNM stage thus providing an excellent tool for further stratification of patients in the same stage and allowing risk-adapted therapy in individual patients.

## Figures and Tables

**Table 1:** Clinical studies evaluating the prognostic role of MTV and TLG measured in primary tumors of NSCLC patients**.**

Clinical Study	N° of Patients	TNM Stage	Endpoints	Volumetric Parameters	Threshold or Delineation Method	Determination of Cut-Off Value	Cut-Off Values	
							MTV	TLG
Davison et al. (2013) [47]	39	I/IV	12 mo. Survival OS	MTV/TLG	gradient-based	median value ROC curve	9.7 mL 79 mL	74 g 349 g
Hyun et al. (2013) [40]	529	IA/IIB	OS/DFS	MTV/TLG	mediastinal background SUVavg plus its 2 SD	ROC curve	16 cm^3^	70 g
Anwar et al. (2018) [48]	49	IA/IB	DFS	MTV/TLG	SUV (2.5)	ROC curve	6.6 mL	36.6 g
Dosani et al. (2019) [49]	134	inoperable IA/IB	LC OS	MTV/TLG	gradient-based	median value	2.4 mL	10.9 g
Yanarates et al. (2020) [50]	258	IIIB/IV	OS/PFS	MTV/TLG	50%SUVmax	ROC curve	5.7 mL	49.4 g
Kim et al. (2014) [51]	63	IA/IIB	OLM	MTV/TLG	SUV (2.5)	ROC curve	18.9 cm^3^	88.4 g
Park et al. (2015) [52]	139	I	OLM	MTV/TLG	SUV (2.0)	ROC curve	3.055 mL	9.829 g
Roengvoraphoj et al. (2018) [53]	65	inoperable IIIA/IIIB	OS	MTV	50%SUVmax	pre-CRT post-CRT Δmid-CRT	63 cm^3^ 25 cm^3^ ≥15%	- - -
Roengvoraphoj et al. (2018) [54]	60	inoperable IIIA/IIIB	OS	MTV	50%SUVmax	Δpost-CRT	≥80%	-

MTV metabolic tumor volume; TLG total lesion glycolysis; NSCLC non-small cell lung cancer; OS overall survival; ROC receiver operating characteristic; DFS disease free survival; SUV standardized uptake value; SD standard deviation; LC local control; PFS progression-free survival; OLM occult lymph node metastasis; CRT chemoradiotherapy.

**Table 2:** Clinical studies evaluating the prognostic role of whole-body MTV and TLG of NSCLC patients**.**

Clinical Study	N° of Patients	TNM Stage	Endpoints	Volumetric Parameters	Threshold or Delineation Method	Determination of Cut-Off Value	Cut-Off Values	
							MTV	TLG
Bazan et al. (2017) [57]	230	inoperable IIB/IIIB	OS LC	MTV	60% adaptive threshold of the SUVpeak within each lesion	median value	32 mL	-
Finkle et al. (2017) [58]	330	IIB/IIIB	OS	MTV	gradient-based	Log-rank test	29.2 mL	-
Ventura et al. (2020) [59]	193	operable I/IV	OS	MTV/TLG	42%SUVmax	ROC curve	8.15 mL	21.85 g
Liao et al. (2012) [24]	169	inoperable lI/IV	OS	MTV/TLG	gradient-based	tertiles	33.5 mL 134.9 mL 473.0 mL	107.3 g 504.0 g 1898.1 g
Pellegrino et al. (2019) [60]	65	I/IV	OS/PFS	MTV/TLG	SUV (2.5)	ROC curve	9.5 mL	54.7 g
Chen et al. (2012) [61]	105	I/IV	OS/PFS	TLG	50%SUVmax	ROC curve	-	655 g
Vanhove et al. (2018) [62]	105	I/IV	OS/PFS	MTV/TLG	50%SUVmax	median value	14.6 mL	93.4 g
Lapa et al. (2017) [63]	278	I/IV	OS	MTV	SUV (2.5)	R software	49.5 mL	-
Pu et al. (2018) [64]	935	I/IV	OS	MTV	gradient-based	quartiles	10 mL 53.4 mL 155 mL	- - -
Chin et al. (2018) [65]	55	oligometastatic I/IV	OS	MTV/TLG	gradient-based	quartiles (highest vs. remaining)	17.8 mL	86.8 g
Kong et al. (2019) [29]	102	inoperable I/III	OS	MTV/TLG	Auto-segmentation at tumor/aorta ratio of 1.5 followed by manual editing according to CT anatomy	median value after mid-RT with conventional RT or PET-adapted RT	41 mL 46 mL	- -
Chen et al. (2019) [66]	25	IIIA/IIIB	OS/PFS	MTV/TLG	50%SUVmax	Δmedian value after mid-RT	42%	65%
Xiao et al. (2017) [67]	17	II/III	RT adjustment based on ΔMTV	MTV	fixed source/background ratio combined with CT anatomy based manual editing	mean value pre-RT mean value during-RT	136.2 mL 64.7 mL	- -

MTV metabolic tumor volume; TLG total lesion glycolysis; NSCLC non-small cell lung cancer; OS overall survival; LC local control; SUV standardized uptake value; ROC receiver operating characteristic; PFS progression-free survival; CT computed tomography; RT radiotherapy; PET positron emission tomography.

## Data Availability

Not applicable.
